# Identification of prognostic signature in cancer based on DNA methylation interaction network

**DOI:** 10.1186/s12920-017-0307-9

**Published:** 2017-12-21

**Authors:** Wei-Lin Hu, Xiong-Hui Zhou

**Affiliations:** 1 College of Informatics, Huazhong Agricultural University, Wuhan, 430070 People’s Republic of China; 2 College of Science, Huazhong Agricultural University, Wuhan, 430070 People’s Republic of China

**Keywords:** DNA methylation interaction network, Biomarker, Cancer prognosis, Systems biology

## Abstract

**Background:**

The identification of prognostic biomarkers for cancer patients is essential for cancer research. These days, DNA methylation has been proved to be associated with cancer prognosis. However, there are few methods which identify the prognostic markers based on DNA methylation data systematically, especially considering the interaction among DNA methylation sites.

**Methods:**

In this paper, we first evaluated the stabilities of microRNA, mRNA, and DNA methylation data in prognosis of cancer. After that, a rank-based method was applied to construct a DNA methylation interaction network. In this network, nodes with the largest degrees (10% of all the nodes) were selected as hubs. Cox regression was applied to select the hubs as prognostic signature. In this prognostic signature, DNA methylation levels of each DNA methylation site are correlated with the outcomes of cancer patients. After obtaining these prognostic genes, we performed the survival analysis in the training group and the test group to verify the reliability of these genes.

**Results:**

We applied our method in three cancers (ovarian cancer, breast cancer and Glioblastoma Multiforme).

In all the three cancers, there are more common ones of prognostic genes selected from different samples in DNA methylation data, compared with gene expression data and miRNA expression data, which indicates the DNA methylation data may be more stable in cancer prognosis. Power-law distribution fitting suggests that the DNA methylation interaction networks are scale-free. And the hubs selected from the three networks are all enriched by cancer related pathways. The gene signatures were obtained for the three cancers respectively, and survival analysis shows they can distinguish the outcomes of tumor patients in both the training data sets and test data sets, which outperformed the control signatures.

**Conclusions:**

A computational method was proposed to construct DNA methylation interaction network and this network could be used to select prognostic signatures in cancer.

**Electronic supplementary material:**

The online version of this article (10.1186/s12920-017-0307-9) contains supplementary material, which is available to authorized users.

## Background

Cancer prognosis is of great research value, but also a huge challenge for today’s medical research [1]. Cancer patients are often over-treated because it is difficult to distinguish low-risk cancer patients from high-risk ones [2]. These days, with the development of the high throughout data of disease samples, it is familiar to select prognostic genes in cancer using gene expression data or other high throughout data [3–5]. These prognostic genes can be used to guide the treatment of cancer patients [6], and they may be candidates of targets for cancer therapy [7]. However, most of the signatures provided by previous work couldn’t perform well in other data sets [8], resulting from the heterogeneity of tumor [9]. Therefore, most of the selected genes may be passengers instead of drivers [9].

In the meanwhile, DNA methylation data, which can reflect the influence of external factors (such as infection and smoking) to the patients [10, 11], is considered as a rising star in the field of cancer research [12, 13]. DNA methylation is a primary epigenetic modified form of genomic DNA, which is an important means of regulating genomic function [14]. DNA methylation data can be used to screen genes that play a key role in the development, progression and metastasis of cancer [15–18]. In addition, there are also a few methods identifying the prognostic genes based on DNA methylation data. For example, Sandoval et al. proposed a DNA methylation signature for prognosis in non-small-cell lung cancer [19], and Lasseigne et al. identified novel diagnostic biomarkers in renal cell carcinoma using DNA methylation profiling [20].

As we know, cancer is a complex polygenic disease, and the occurrence of cancer is usually caused by the role of several genes. Network biology, which applies biological network to describe the relationship among genes, could be applied to study the complex diseases [21, 22]. Therefore, it is a promising solution to prioritize the biomarkers of cancer prognosis through biological networks. There are also a few works applying DNA methylation co-expression network to understand biological systems [23, 24]. However, as far as we know, there are few works considering the DNA methylation networks to select the prognostic signatures in cancer systematically.

Breast cancer (BRCA) and Ovarian cancer (OV) are the most popular cancers in women [25], and glioblastoma multiforme (GBM) is a fast-growing type of malignant brain tumor that is the most common brain tumor in adults [26]. Therefore, it is urgent to identify the prognostic genes in these cancers [27–30]. Based on the hypothesis that the co-expression relation among the DNA methylation sites may reveal the interaction among the according genes in some aspects, we identify the prognostic genes of ovarian cancer, breast cancer and glioblastoma multiforme using DNA methylation interaction networks respectively. Firstly, using matched gene expression data, miRNA expression data and DNA methylation data of tumor samples from TCGA (The Cancer Genome Atlas), we evaluated the stability of the three kinds of data when they were used in cancer prognosis. Secondly, DNA methylation interaction network was constructed by considering the co-expression among all the DNA methylation sites of the whole genome. Thirdly, topological analysis of the network was performed to check whether our network had the similar topological characteristics of biological network. Fourthly, functions of the hubs in the network were investigated to check whether our network could reveal the biological mechanism of cancer. Fifthly, we used Cox regression to select the hubs whose DNA methylation levels were significantly correlated to cancer patients’ outcome, and these hubs were set as prognostic genes. Finally, the prognostic signatures were evaluated by survival analysis.

## Methods

### Data sets

TCGA [31] provides high-throughput sequencing data of genomes, as well as clinical data of a variety of tumor samples. Here, we downloaded the data sets of ovarian cancer, breast cancer and glioblastoma multiforme, including the clinical data (days to death, state of death), DNA methylation data (JHU-USC HumanMethylation 450, level 3), miRNA expression profiles (Agilent 8 × 15 K Human miRNA-specific microarray, level 3) and gene expression profiles (UNC Agilent G4502A_07, level 3) of cancer patients. As to the DNA methylation data, there are 605 samples in ovarian cancer, 343 samples in breast cancer, and 295 samples in glioblastoma multiforme respectively. The DNA methylation data of all the samples was applied to construct the DNA methylation network. And only the samples with matched DNA methylation data, mRNA expression data and miRNA expression data were used to evaluate the stability of the three types of data. When the data was applied in survival analysis, the samples with mapped clinical information and DNA methylation data were used.

In the level 3 data of DNA methylation data, each methylation site was mapped to one gene for most DNA methylation sites. Of course, there are also some DNA methylation sites mapped to two genes. In this work, we used the DNA methylation sites to construct the network and identify the features for cancer prognosis. When we investigate the functions of the nodes with the highest degrees in the network, the DNA methylation sites were mapped to genes. Except for that, in all the analysis, methylation levels of the DNA methylation sites were used.

### Construction of DNA methylation interaction network

Since the DNA methylation levels of the DNA methylation sites do not obey the normal distribution in the cancer samples, we applied Spearman rank correlation, which is a nonparametric test, to calculate the correlation coefficient of the DNA methylation levels between every two DNA methylation sites. The Spearman rank correlation is calculated as follows:

1 $$ \rho =1-\frac{6\sum \limits_{\mathrm{i}=1}^{\mathrm{n}}{d}_i^2}{n^3-n}. $$

Here, n is the number of cancer samples in the cancer data set. For each DNA methylation site, it is sorted from large to small based on the DNA methylation value in the n cancer samples. *d*_*i*_ is the difference of a certain gene pair calculated by the sort value of the DNA methylation value in the i-th cancer sample. Thus, if the sorting of DNA methylation values of DNA methylation sites are the same in all cancer patients (from large to small), then the correlation coefficient is 1. Through this calculation, we can calculate the correlation coefficient between any pair of genes.

Based on the Spearman correlation coefficient, we constructed our DNA methylation interaction network using a simple rank-based method which is proposed by Ruan [32]. As we know, value-based methods are significantly limited because there is a homogeneous threshold for all the genes in the network, which means some genes are almost significantly related to almost all genes (such as house-keeping genes), while some gene pairs will be excluded from the network because their *p*-value is higher than the threshold though they do have certain kinds of significant connection. In fact, genes in one functional pathway may be strongly mutually co-expressed, while genes in another functional pathway may be only weakly co-expressed [32]. Therefore, applying the similar strategy of the rank-based method [32], for each site we selected only 10 most relevant sites as its neighbors, so that all selected pairs of DNA methylation sites constituted a DNA methylation interaction network.

### The selection of prognostic genes in ovarian cancer

Based on the hypothesis that if a gene is located in the hubs of the DNA methylation interaction network, the gene can be good candidates for cancer prognosis, the prognostic signatures of the three cancers were selected as follow:

Firstly, the top 10% genes (DNA methylation sites) with the largest degrees were selected as hub genes.

Secondly, univariable Cox regression was applied to screen out the hubs whose DNA methylation levels were significantly related to the outcome of patients. In this regression model, independent variable is the DNA methylation levels of each DNA methylation site across all the patients, and dependent variable is the prognostic risks (death time as well as status of death) of all the patients. Finally, the significant hubs were selected as prognostic genes in the corresponding cancer.

### Evaluation of the prognostic signatures

After obtaining the prognostic features of cancer patients, we used a strategy similar to Gene expression Grade Index (GGI) [33] to predict the death risk for every cancer sample:

2 $$ \mathrm{PrognosisRisk}=\sum {p}_i-\sum {q}_j. $$

Here, *p*_*i*_ is the DNA methylation level of genes with positive Cox coefficient, and *q*_*j*_ is the DNA methylation level of genes with negative Cox coefficient. Then the samples, whose risk scores were among the top 50%, were divided into the bad-outcome group, and the other ones were divided into the good-outcome group. In the end, the log rank test was performed to test the difference of the patients’ overall survival between the two classes.

### Enrichment analysis

We used GSEA [34] to perform function annotation for the hub genes. And we used hypergeometric test to investigate whether the overlap of selected genes from different data set is significant. This test is shown as follow:

3 $$ \mathrm{p}- value=1-\sum \limits_{i=0}^{x-1}\frac{\left({C}_K^i\times {C}_{M-K}^{N-i}\right)}{C_M^N}. $$

Here, x describes the number of genes of the overlap; K and N describe the number of selected features from one data set and the other data set respectively, and M is the number of the genes in the universal set.

### Network visualization and analysis

Cytoscape 3.5.1 was used to visualize our DNA methylation interaction network. In addition, we used a plug-in in Cytoscape to analyze the network [35].

## Results

### DNA methylation data is more stable in cancer prognosis

**Fig. 1:**
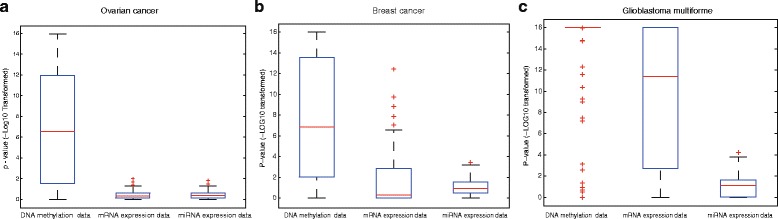
Comparision of the stabilities of DNA methylation, mRNA expression and miRNA expression data. The stabilities were evaluated by the overlap of the prognostic genes selected from different samples. The significances of the overlaps were calculated by hypergeometric distribution test. **a** The evaluation result in ovarian cancer data set. **b** The evaluation result in breast cancer data set. **c** The evaluation result in glioblastoma multiforme

As we know, the biggest problem of the prognostic genes identified based on high-throughput data is the lack of stability. For example, 76 prognostic genes and 70 prognostic genes were both identified for prognosis in breast tumor using mRNA expression profiles. Both of the prognosis models performed well in their own data sets. However, their performances in independent data sets were poor [36]. In addition, there is little overlap between the two signatures [37]. So we first systematically evaluated the stabilities of microRNA expression value, gene expression value, and DNA methylation data for each of the three cancers respectively. First of all, in the TCGA cancer data set, the same number of samples was randomly assigned into two groups. And then respectively in both groups, we applied Cox regression to select the genes (or miRNA) whose expression levels were significant related to the prognosis of tumor samples (*p*-value <0.05). After that, we performed hypergeometric distribution test to assess whether the overlap of the two sets selected from the two groups was significant. After repeating the above steps for 100 times, we obtained 100 *p*-values (hypergeometric distribution test) for each of the three kinds of data, which is shown in Fig. 1.

We can see that these selected genes from DNA methylation data are more stable in all the three cancers. That is, the overlaps of the genes selected from different samples based on DNA methylation data are more significant. Furthermore, Wilcoxon rank sum test was applied to test the differences between the p-values in DNA methylation data and other two kinds of data. *P*-values of the Wilcoxon rank sum test are 1.10E-20 and 3.54E-20 in ovarian cancer respectively. In breast cancer, the p-value of Wilcoxon rank sum test of the stabilities between the DNA methylation data and the mRNA expression data (miRNA expression data) is 2.06E-13 (6.34E-15). The similar result could be found in the data set of glioblastoma multiforme, the p-value of the test of stability between the DNA methylation data and the mRNA expression data (miRNA expression data) is 8.42E-08 (3.96E-29). From all these results, it was concluded that in the aspect of prognosis of the three cancers, compared with mRNA and microRNA data, DNA methylation data may be more stable. As we know, the main problem in cancer prognosis is that the gene signature lacks stability. Therefore, DNA methylation data may be promising to prioritize prognostic signature.

### The DNA-methylation interaction network

As the co-expression of the DNA methylation sites may reveal the interaction of the according genes in some aspects, we used DNA methylation data to construct the DNA methylation interaction networks using three cancer data sets in TCGA (Method) respectively. In this work, we adopted a rank-based method [32] to solve this problem.

**Fig. 2:**
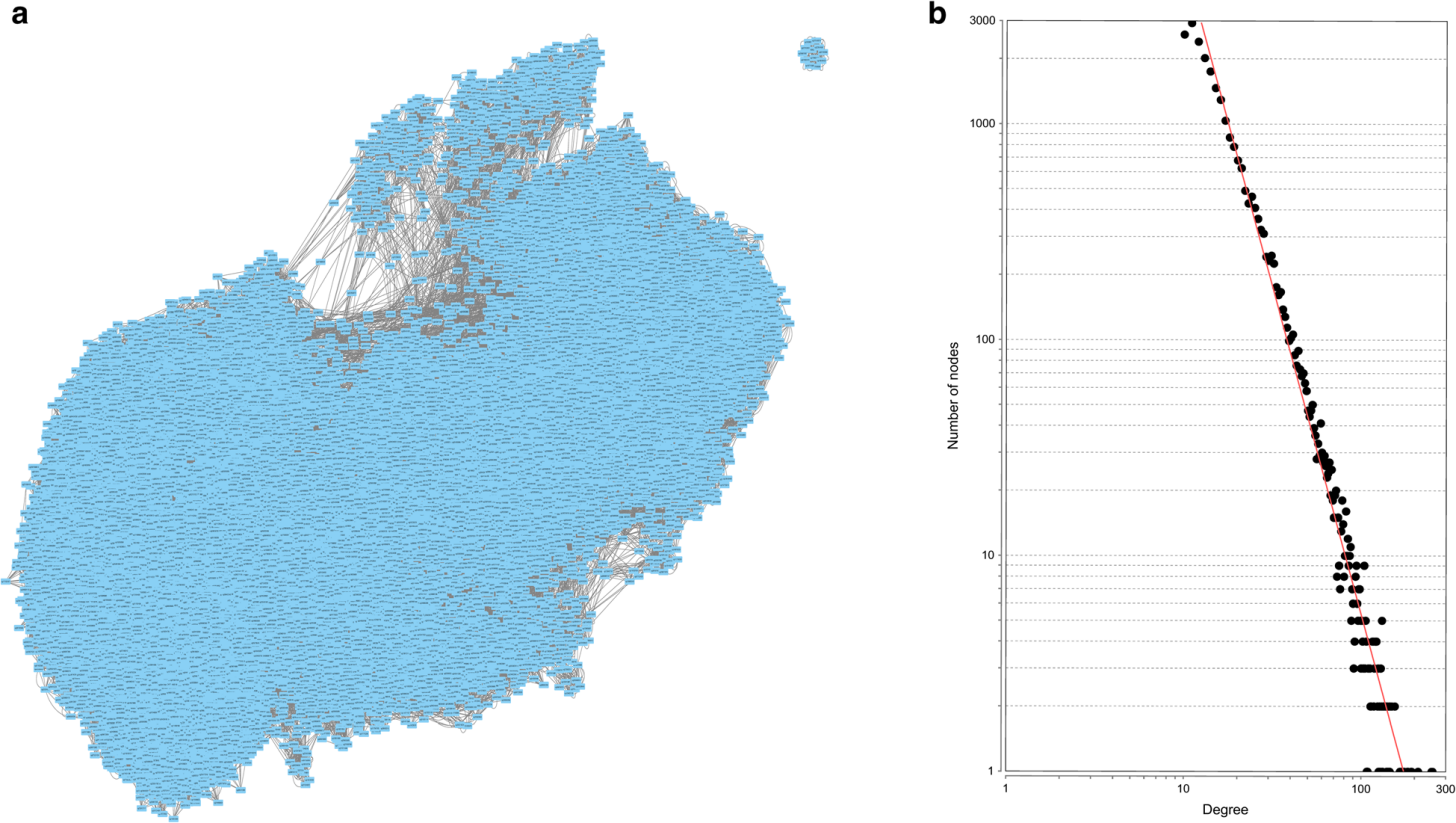
The DNA methylation interaction network of ovarian cancer. **a** Overview of the DNA methylation interaction network. **b** The power-law fitting of the degrees of the nodes in the network

In ovarian cancer data set, we obtained 249,810 significant pairs among 24,981 nodes, which are shown in Fig. 2a (Additional file 1: Table S1). In this network, an edge between two nodes describes that DNA methylation levels of the two sites is correlated. In addition, the nodes’ degrees fits well with the power-law distributions, and the correlation and R-square of the fittings are 0.964 and 0.969 (Fig. 2b), which indicates the network is scale free. As we know, the scale-free network conforms to the biological network’s topological characteristics.

In breast cancer data set, the DNA methylation interaction network is shown in Additional file 2: Figure S1 and all the significant pairs are shown in Additional file 3: Table S2. Power-law fit was also applied to investigate the topological characteristics of the network. As a result, the correlation is 0.982 and R-square is 0.926 (Additional file 2: Figure S2), which indicates the DNA methylation interaction network in breast cancer is also scale free.

The DNA methylation interaction network of glioblastoma multiforme is shown in Additional file 2: Figure S3 and all the significant pairs are shown in Additional file 4: Table S3. Being similar with networks of ovarian cancer and breast cancer, the degrees of the nodes in the network of glioblastoma multiforme also follows the power-law distributions, with its correlation of 0.998 and R-square of 0.953 (Additional file 2: Figure S4).

As we know, the hubs in the scale-free network may play important roles [38]. Therefore, in the DNA methylation interaction network which is constructed using cancer data set, a node with a large degree may be more essential in the biological processes of cancer. Therefore, it is expected that the hubs in our network may be more likely to affect the prognosis of the cancer. In this work, we selected approximately 10% of all the nodes in the network as hubs for our next analysis. As a result, 2502 genes with the highest degree (no less than 35) in the network of ovarian cancer (Additional file 5: Table S4), 2509 genes (with the degree of no less than 34) in the network of breast cancer (Additional file 6: Table S5) and 2584 genes (with the degree of no less than 35) in the network of glioblastoma multiforme (Additional file 7: Table S6) were obtained respectively.

### Functional annotation of the hub genes

We used GSEA [34] to analyze which pathways the hub genes are involving in. The enriched KEGG pathways for hubs in the DNA methylation interaction network of ovarian cancer are shown in Table 1.

**Table 1:** Functional annotation of the hub genes in ovarian cancer

Pathways	*p*-value	FDR q-value
Pathways in cancer	2.89E-24	5.37E-22
MAPK signaling pathway	4.17E-16	3.88E-14
Wnt signaling pathway	1.23E-13	7.62E-12
p53 signaling pathway	5.23E-13	2.43E-11
Prostate cancer	3.97E-12	1.48E-10
Cell cycle	4.97E-12	1.54E-10
Endocytosis	9.75E-12	2.59E-10
Neurotrophin signaling pathway	1.81E-11	4.20E-10
Apoptosis	2.12E-11	4.38E-10
Small cell lung cancer	5.05E-11	9.40E-10
Leishmania infection	9.37E-11	1.58E-09
Cytokine-cytokine receptor interaction	3.45E-10	5.35E-09
Regulation of actin cytoskeleton	1.24E-09	1.77E-08
Purine metabolism	1.43E-09	1.90E-08
Jak-STAT signaling pathway	3.27E-09	4.06E-08
Ubiquitin mediated proteolysis	4.10E-09	4.77E-08
Toll-like receptor signaling pathway	8.42E-08	9.21E-07
Focal adhesion	1.06E-07	1.09E-06
Pyrimidine metabolism	1.98E-07	1.93E-06
Glycosphingolipid biosynthesis - lacto and neolacto series	4.40E-07	4.09E-06
Amyotrophic lateral sclerosis (ALS)	4.85E-07	4.29E-06
Neuroactive ligand-receptor interaction	5.23E-07	4.42E-06
T cell receptor signaling pathway	1.02E-06	8.22E-06
Alzheimer’s disease	1.14E-06	8.77E-06
Chronic myeloid leukemia	1.18E-06	8.77E-06
Phosphatidylinositol signaling system	2.08E-06	1.49E-05
Tight junction	2.48E-06	1.71E-05
NOD-like receptor signaling pathway	3.73E-06	2.47E-05
Non-small cell lung cancer	3.85E-06	2.47E-05
Melanoma	4.11E-06	2.47E-05
Spliceosome	4.21E-06	2.47E-05
Leukocyte transendothelial migration	4.24E-06	2.47E-05
Axon guidance	4.78E-06	2.69E-05
Lysosome	6.29E-06	3.44E-05
Glioma	6.73E-06	3.58E-05
B cell receptor signaling pathway	8.37E-06	4.33E-05
Oocyte meiosis	9.39E-06	4.72E-05
Base excision repair	9.77E-06	4.73E-05
VEGF signaling pathway	9.92E-06	4.73E-05
Epithelial cell signaling in Helicobacter pylori infection	1.17E-05	5.44E-05
Endometrial cancer	1.44E-05	6.53E-05
Colorectal cancer	1.94E-05	8.58E-05

The *p*-value and FDR q-value was provided by GSEA, which was applied to evaluate the significance of the enrichment analysis

Among the 42 significant pathways (FDR < 1.0E-4), the most impressive one is ‘Pathways in cancer’, which is the most significant pathway with an FDR of 5.37E-22. What’s more, many sub-pathways of ‘Pathways in cancer’ were enriched, such as ‘MAPK signaling pathway’, ‘Wnt signaling pathway’, ‘p53 signaling pathway’, ‘Apoptosis’, ‘Jak-STAT signaling pathway’, ‘Cytokine-cytokine receptor interaction’, ‘Focal adhesion’ and ‘VEGF signaling pathway’. In these sub-pathways, ‘MAPK signaling pathway’ is reported to be essential for cancer-immune evasion in human cancer cells [39]. What’s more, ‘Wnt signaling pathway’ is validated to be able to cause cancer [40]. ‘p53 signaling pathway’, one of the most famous cancer related pathways, is also known for its potentially universal involvement in the etiology of cancer [41]. Apart from this, there were also many pathways in specific cancers involved, such as ‘Small cell lung cancer’, ‘Prostate cancer’, ‘endometrial cancer’ and ‘colorectal cancer’.

The enriched pathways for hub genes in breast cancer are shown in Additional file 8: Table S7. A total of 18 pathways are significant with FDR less than 1.0E-04. In addition, the most significant one is ‘Pathway in cancer’ (FDR = 2.88E-11). Being similar with those in the ovarian cancer, some sub-pathways of ‘Pathway in cancer’ were obtained by the enrichment analysis, such as MAPK signaling pathway, Focal adhesion, Wnt signaling pathway and Cytokine-cytokine receptor interaction. However, there were also some cardiomyopathy related pathways significant (‘Hypertrophic cardiomyopathy (HCM)’, ‘Arrhythmogenic right ventricular cardiomyopathy (ARVC)’), which was different from the enrichment pattern of the hubs in the network of ovarian cancer. In the meanwhile, cardiomyopathy is a common side effect of breast cancer treatment [42, 43].

As to the hubs in the network of glioblastoma multiforme, 60 pathways were enriched with a FDR less than 1.0E-04 (Additional file 9: Table S8). The enrichment pattern is similar with those of the breast cancer and ovarian cancer. The most significant pathway is ‘Pathway in cancer’ (FDR = 1.85E-13), and some sub-pathways of ‘Pathway in cacner’ are also significant, such as MAPK signaling pathway, Wnt signaling pathway, Apoptosis, p53 signaling pathway and PPAR signaling pathway. What’s more, the hub genes were directly enriched by ‘Glioma’, with a FDR of 3.39E-07. Apart from that, some Drug metabolism related pathways were also significant. For example, ‘Drug metabolism - cytochrome P450’ is significant with a FDR of 7.76E-05. As we know, it is a very important drug metabolism pathways for anti-cancer drug [44], and it is related to the drug response to cancer patients [45].

In a word, our hub genes are significantly enriched by many cancer-related pathways.

### Prognostic genes selected from hubs

As we mentioned above, the hub node in the DNA methylation interaction network may be more essential in cancer prognosis. Therefore, the hubs which are significantly related to the outcomes of tumor samples may be good prognostic genes. We randomly divided the samples of ovarian cancer into training group and test group with the same number of samples, which is shown in Additional file 10: Table S9. In the training group, Cox regression was applied to calculate the correlation and *p*-value of each hub’s DNA methylation levels with the prognosis of tumor samples. Finally, 76 DNA methylation sites were selected by a threshold of *p*-value <0.05 (Additional file 11: Table S10). Among the 76 DNA methylation sites, cg02376703 is on the top. That is, among the hubs, cg02376703 is the one of which DNA methylation levels are most related to the prognosis of ovarian-cancer patients. It was annotated as two genes: ‘COX8C’ and ‘KIAA1409’. In the meanwhile, the alterations of COX8C is associated with epithelial ovarian cancer risk [46] and KIAA1409 is a tumor suppressor gene [47].

Based on the training data set of breast cancer in TCGA (Additional file 12: Table S11), 69 DNA methylation sites, whose DNA methylation levels were significantly related to the prognosis of breast tumor samples, were selected as the prognostic signature in breast cancer (Additional file 13: Table S12). Among these sites, cg05142115 (Gene Symbol: USP10) is the most significant one. P53 is the most famous tumor suppressor gene [48] and it is reported that USP10 is regulator of p53, providing an alternative mechanism of p53 inhibition in cancers with wild-type p53 [49].

In glioblastoma multiforme, 88 hubs, whose DNA methylation levels were significantly related to the prognosis of tumor samples in the training data set of glioblastoma multiforme in TCGA (Additional file 14: Table S13), were selected as the gene signature of glioblastoma multiforme (Additional file 15: Table S14). The most significant one (cg19465374: PODXL) was also investigated by literature survey. And it is reported that high PODXL expression is related to increasing glioma grade and decreased survival time in patients with glioblastoma multiforme [50].

In summary, 76 hubs in ovarian cancer, 69 hubs in breast cancer and 88 hubs in glioblastoma multiforme were selected based on the corresponding DNA methylation data and DNA methylation interaction networks. Case studies of the most significant ones of the prognostic genes showed they were indeed cancer related.

### Evaluation the prognosis signatures by survival analysis

As we know, the main problem of the prognosis signatures based on mRNA expression profiles is poorly generalized [51]. With the purpose of evaluating our signature, the selected genes were applied to calculate the risk score of tumor samples in both the training data set and test data set for the three cancers respectively (Method). For comparison, we also applied Cox regression to pick out those sites of which DNA methylation levels are most related to the prognosis of tumor samples as control signature.

**Fig. 3:**
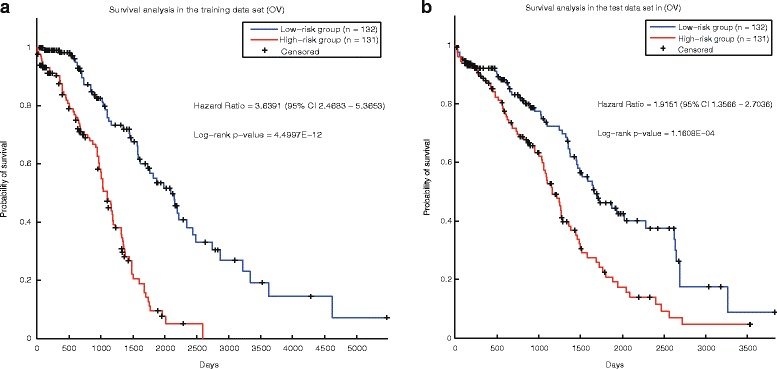
Survival analysis of the ovarian cancer samples stratified by our signature. **a** The training set. **b** The test set

In ovarian cancer, survival analysis of the samples in the two groups classified by our prognostic genes in the training data set shows these genes can distinguish the prognosis of tumor samples, with a HR (hazard ratio) of 3.64 (95% CI 2.47–5.37). And log-rank *p*-value of the overall survival between these patients in the two classes is 4.50E-12 (Fig. 3a). Furthermore, we evaluated our method in the test data set. HR of the overall survival of the patients in the two groups divided by the prognostic signature is 1.92 (95%CI 1.36–2.70), and the corresponding p-value is 1.16E-04 (Fig. 3b). For comparison, we also used the control signature (Additional file 16: Table S15) to calculate the risk scores of tumor samples in the same data sets. The control signature can also stratify the patients of the training data set into different-prognosis groups, with the HR and *p*-value of 3.90 and 8.66E-15 (Additional file 2: Figure S5.a). This is not strange because the control genes are the most significant genes which are selected in the training set. However, in the test set, prognostic risks of the patients divided by the control signature shows no significant difference (Additional file 2: Figure S5.b).

**Fig. 4:**
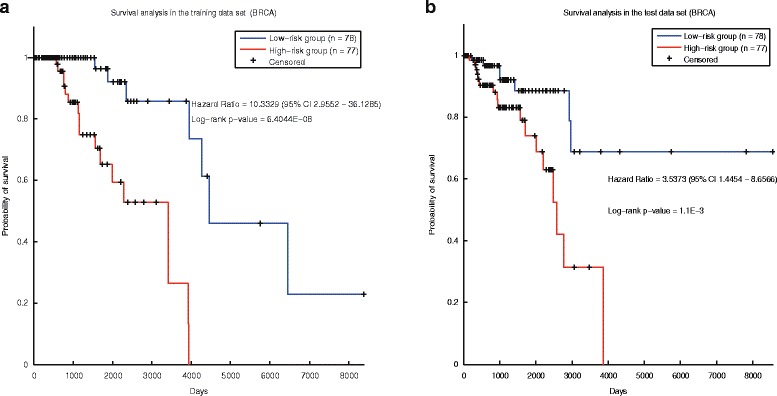
Survival analysis of breast cancer samples stratified by our signature. **a** The training set. **b** The test set

In the breast cancer, based on the 69 prognostic genes (DNA methylation sites), the risk score of the patients could be calculated (Method). In the training data set, the HR of high-risk group and the low-risk group is 10.34, and the p-value is 6.40E-08 (Fig. 4a). Survival analysis in the test data set also shows there are significant difference of the overall survival between the tumor samples of the two classes, with the HR of 3.54 and *p*-value of 1.10E-3 (Fig. 4b). The control signature (Additional file 17: Table S16) was also applied to predict the prognosis of tumor samples in the breast cancer. The control signature performs well in both the training data set (Additional file 2: Figure S6.a) and the test data set (Additional file 2: Figure S6.b). However, our prognostic genes perform better in both data sets.

**Fig. 5:**
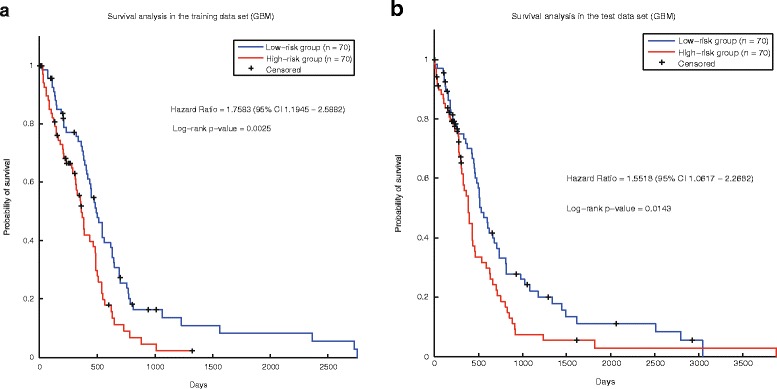
Survival analysis of cancer samples (glioblastoma multiforme) stratified by our signature. **a** The training set. **b** The test set

As to glioblastoma multiforme, the prognostic signature was also applied to calculate the risk score of tumor samples in TCGA GBM. Survival analysis shows that our prognostic signature could distinguish the prognosis of tumor patients in the training set (Fig. 5a) and test data set (Fig. 5b). The HR (p-value) in the two data sets are 1.76 (0.0025) and 1.55 (0.014) respectively. Based on the control signature (Additional file 18: Table S17), the risk scores of the patients were also calculated. The control signature could distinguish the prognosis of tumor samples in the training data set (Additional file 2: Figure S7.a) because the control signature was selected in this data set. However, in the test data set, the control signature performs badly (Additional file 2: Figure S7.b).

From all these results, we can see that the prognostic signature selected by our methods perform well in all the three cancers. In addition, our prognostic signatures outperformed the control signatures. As our prognostic genes were filtered by the essential nodes in the DNA-methylation interaction networks, all these results validated that the DNA-methylation interaction network could facilitate the selection of prognostic signatures.

## Discussion

DNA methylation has been proved to be associated with many biological processes in cancer. However, there are few methods identifying the prognostic markers based on DNA methylation data systematically, especially considering the interaction relationship among DNA methylation sites. Based on the biological hypothesis that DNA-methylation data could reveal the information hidden behind cancer prognosis, and the inter-relationship of DNA methylation sites may reveal biological interactions between genes at a certain level, we used DNA methylation data to construct DNA methylation interaction network, and used this network to identify prognostic genes. We first demonstrated the DNA methylation data may be more stable in cancer prognosis of ovarian cancer, breast cancer and glioblastoma multiforme. After that, we confirmed that the networks were typical scale-free biological networks, and then we used the hub nodes of the DNA methylation interaction networks to perform functional annotation, and the results indicate that they were closely correlated with cancer-related functions. Using the hub nodes, we screened 76 DNA methylation sites in ovarian cacner, 69 DNA methylation sites in breast cancer and 88 DNA methylation sites in glioblastoma multiforme and found that these DNA methylation sites could significantly differentiate the prognostic risks of all the cancer patients.

Here, a computational method was proposed to reconstruct DNA methylation interaction network. This network could be used to select prognostic signatures in cancer. In our opinion, it can also be applied in the study of other biological problem. For example, in the study of disease progress, cell development or any related fields as long as there were enough samples of DNA methylation data.

Of course, some issues of our work should be addressed. (1) Due to the limited availability of cancer data sets of DNA methylation data, our prognostic genes can only be validated in TCGA. (2) As the main purpose of our work is to prove that the DNA methylation interaction network could facilitate the selection of prognostic genes, we only used a naïve prognostic model. However, a more complicated model may make the prognostic model more powerful. We will address these problems in our future work.

## Conclusion

In this work, we have proposed a computational method to construct DNA methylation interaction network. And a pipeline was proposed to identify the prognostic signature in cancer based on the DNA methylation interaction network. Our method was validated in ovarian cancer, breast cancer and glioblastoma multiforme. The experiment results show our prognostic signatures can distinguish the outcome of cancer patients. In addition, these prognostic genes were indeed cancer related.

## Additional files

Additional file 1: Table S1. The DNA methylation interaction network of ovarian cancer. This table describes all the DNA methylation interaction pairs in the network. (XLSX 12331 kb)

Additional file 2: Supplementary manuscript. This file contains the seven supplementary figures and the code for the construction of the DNA methylation interaction network. (DOCX 3703 kb)

Additional file 3: Table S2. The DNA methylation interaction network of breast cancer. This table describes all the DNA methylation interaction pairs in the network. (XLSX 12808 kb)

Additional file 4: Table S3. The DNA methylation interaction network of glioblastoma multiforme. This table describes all the DNA methylation interaction pairs in the network. (XLSX 12818 kb)

Additional file 5: Table S4. Hubs in the network of ovarian cancer. (XLSX 54 kb)

Additional file 6: Table S5. Hubs in the network of breast cancer. (XLSX 57 kb)

Additional file 7: Table S6. Hubs in the network of glioblastoma multiforme. (XLSX 57 kb)

Additional file 8: Table S7. Functional annotation of the hub genes in breast cancer. (XLSX 10 kb)

Additional file 9: Table S8. Functional annotation of the hub genes in glioblastoma multiforme. (XLSX 11 kb)

Additional file 10: Table S9. Samples in the training and test sets of ovarian cancer. (XLSX 16 kb)

Additional file 11: Table S10. The prognostic genes of ovarian cancer. (XLSX 13 kb)

Additional file 12: Table S11. Samples in the training and test sets of breast cancer. (XLSX 14 kb)

Additional file 13: Table S12. The prognostic genes of breast cancer. (XLSX 13 kb)

Additional file 14: Table S13. Samples in the training data set and test data set of glioblastoma multiforme. (XLSX 14 kb)

Additional file 15: Table S14. The prognostic genes of glioblastoma multiforme. (XLSX 14 kb)

Additional file 16: Table S15. The control signature of ovarian cancer. The signature contains the same number of DNA methylation sites with our prognostic genes. The sites in the signature are the most significant ones with the prognostic risks of cancer patients. (XLSX 13 kb)

Additional file 17: Table S16. The control signature of breast cancer. The signature contains the same number of DNA methylation sites with our prognostic genes. The sites in the signature are the most significant ones with the prognostic risks of cancer patients. (XLSX 15 kb)

Additional file 18: Table S17. The control signature of glioblastoma multiforme. The signature contains the same number of DNA methylation sites with our prognostic genes. The sites in the signature are the most significant ones with the prognostic risks of cancer patients. (XLSX 15 kb)
