# Supplementary material for: Identification of prognostic signature in cancer based on DNA methylation interaction network
Source: BMC Med Genomics. 2017 Dec 21;10(Suppl 4):63. doi: 10.1186/s12920-017-0307-9 (PMC5763425; doi:10.1186/s12920-017-0307-9)
Supplement: Supplementary file 2 — Supplementary manuscript. This file contains the seven supplementary figures and the code for the construction of the DNA methylation interaction network. (DOCX 3703 kb) [file 12920_2017_307_MOESM2_ESM.docx]

# Identification of prognostic genes in cancer based on DNA methylation interaction network

Weilin Hu^2^, Xionghui Zhou^1^*

^1^College of Informatics, Huazhong Agricultural University, Wuhan, P. R. China

^2^College of Science, Huazhong Agricultural University, Wuhan, P. R. China

*Correspondence should be addressed to X. Z. (zhouxionghui@mail.hzau.edu.cn).

### This file contains the seven supplementary figures and the matlab code for the construction of the network.


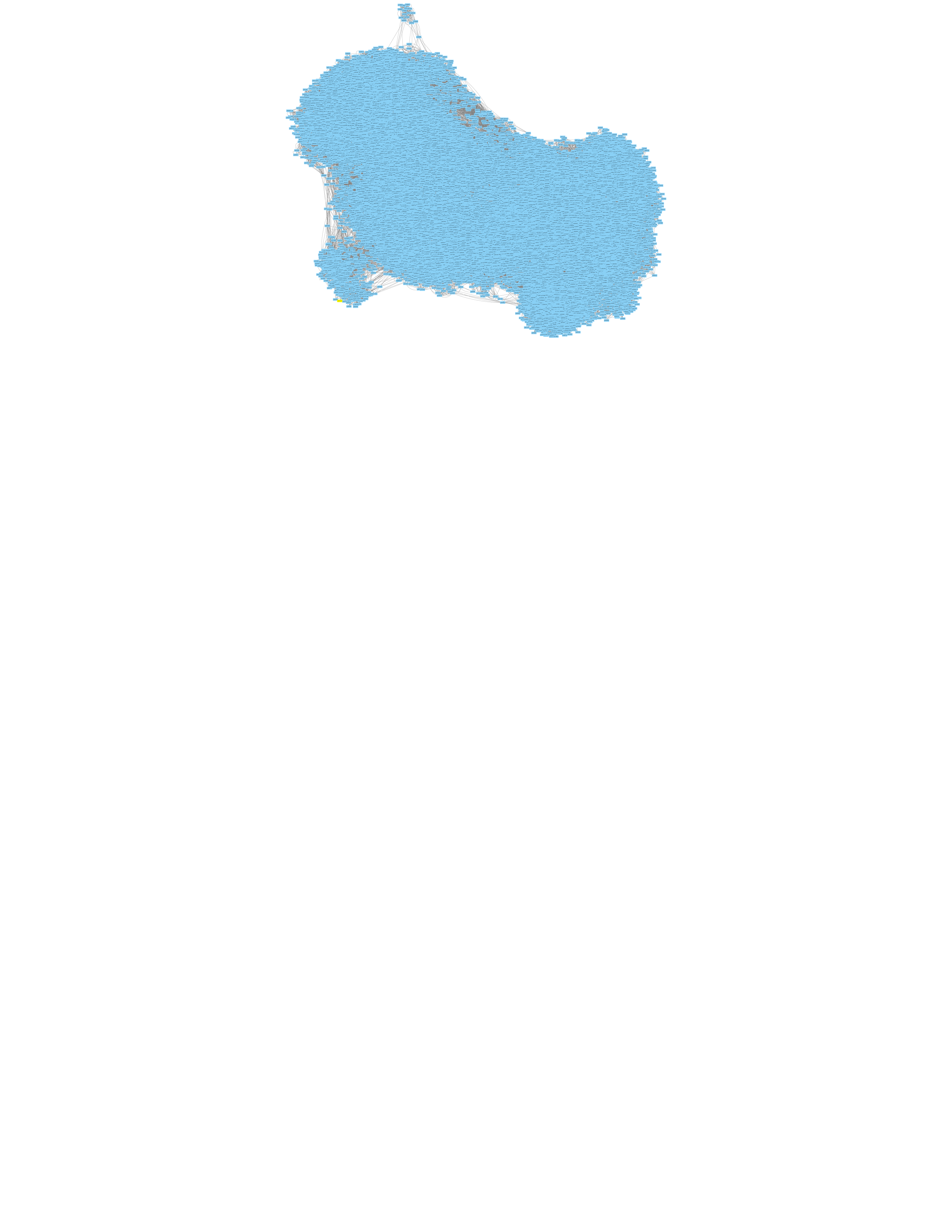


Figure S1. **The DNA methylation interaction network of breast cancer.**

**
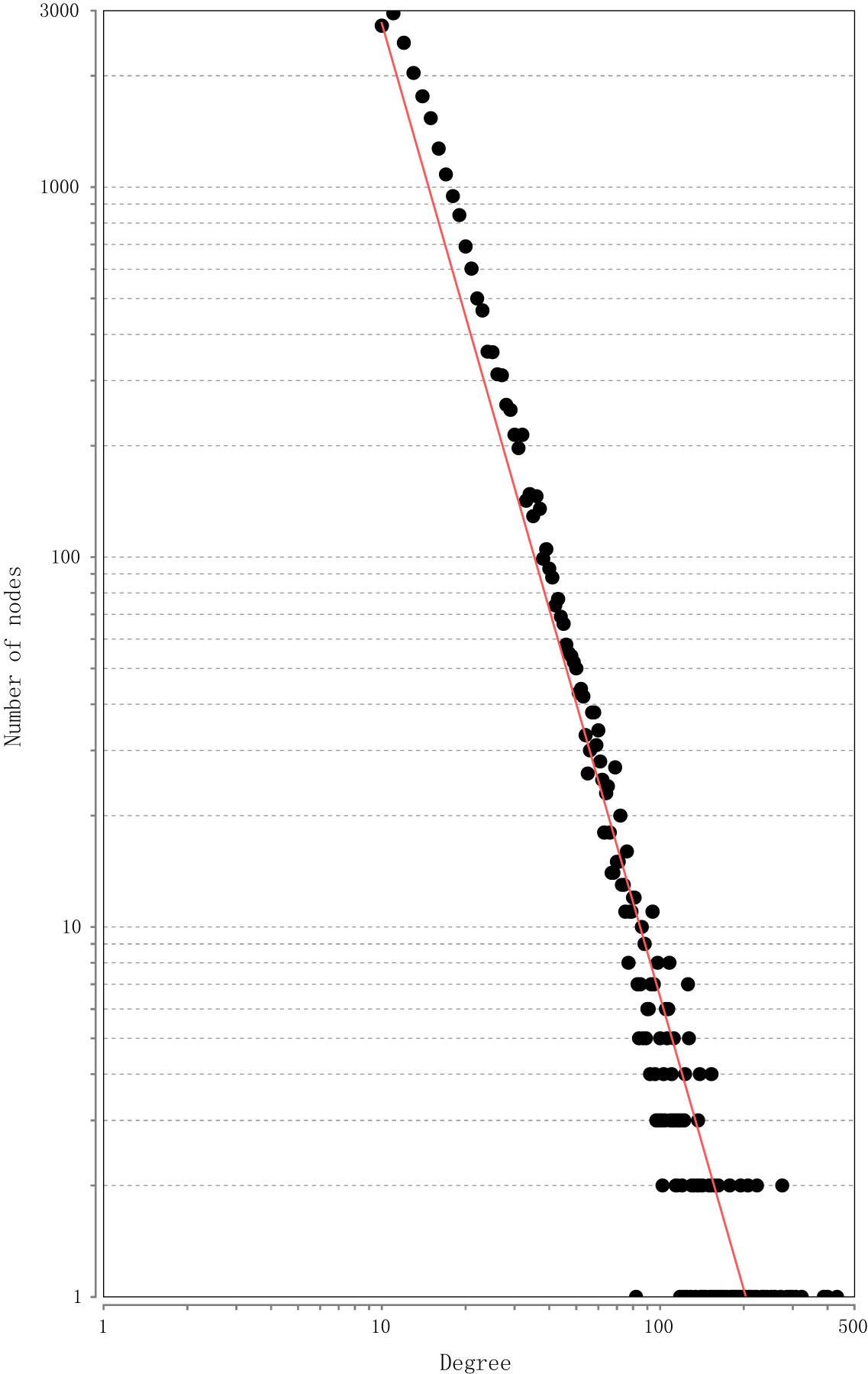
**

Figure S2. **The power law fit of the nodes’ degree in the network of breast cancer.**


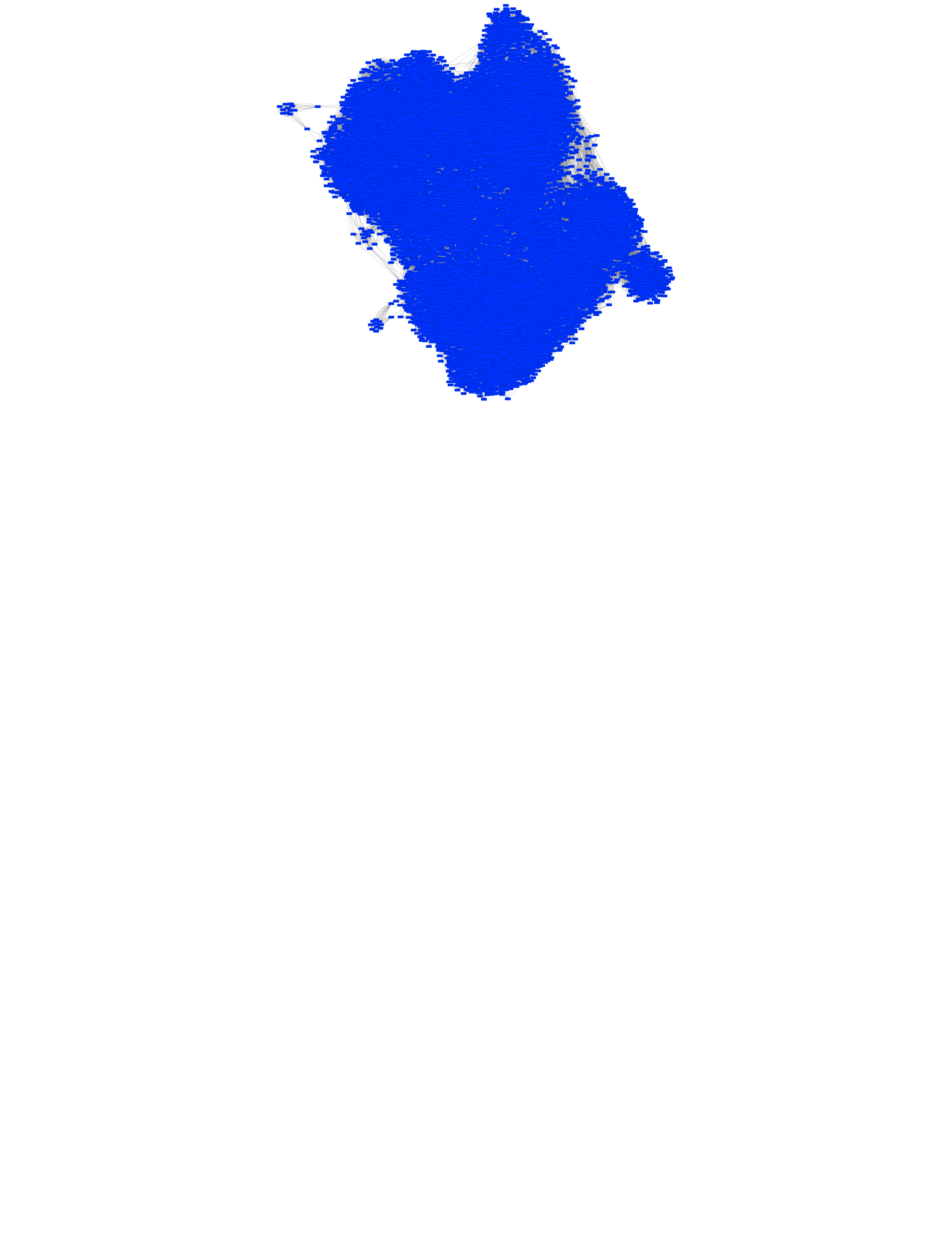


Figure S3. **The DNA methylation interaction network of glioblastoma multiforme.**

**
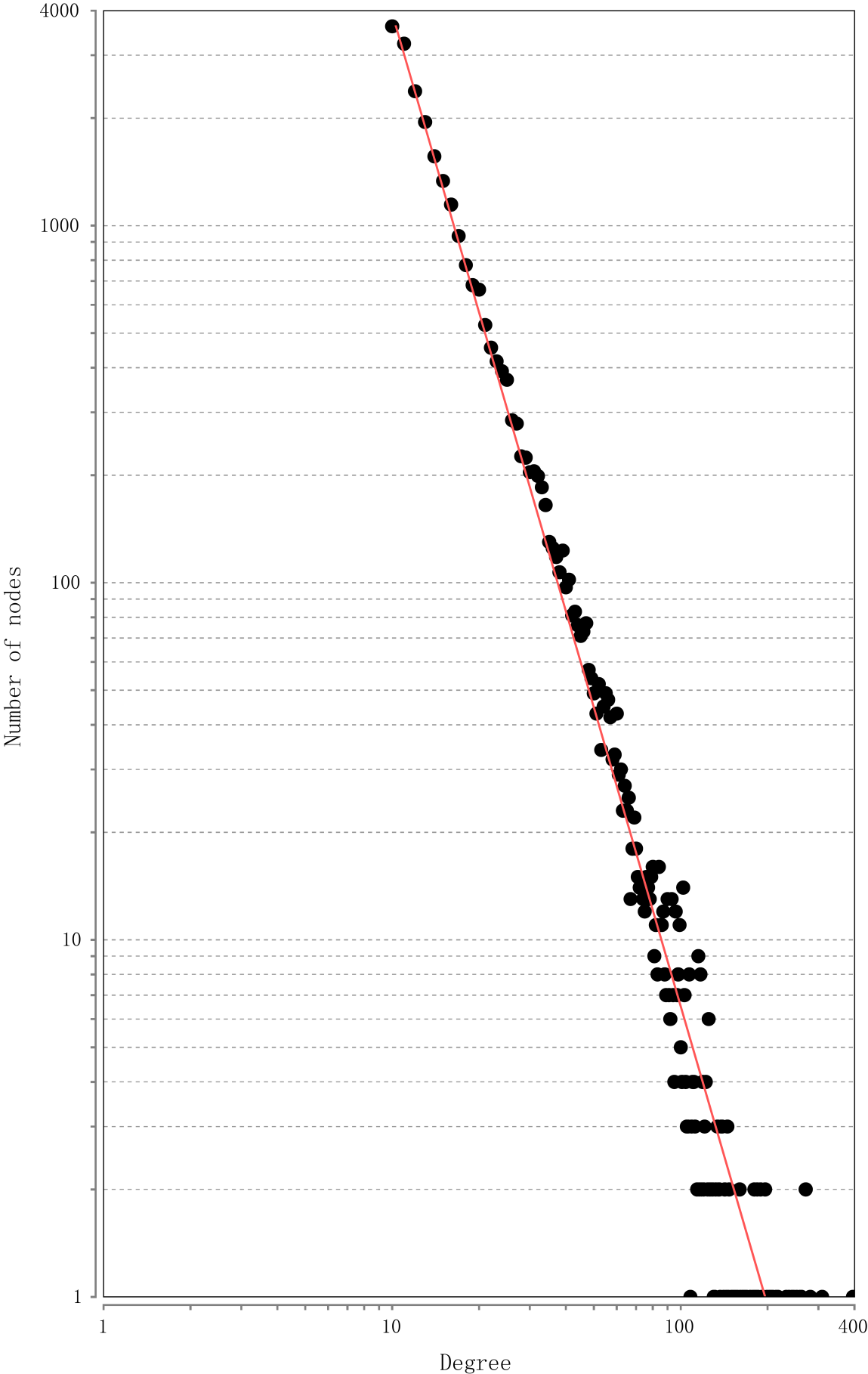
**

Figure S4. **The power law fit of the nodes’ degree in the network of glioblastoma multiforme.**


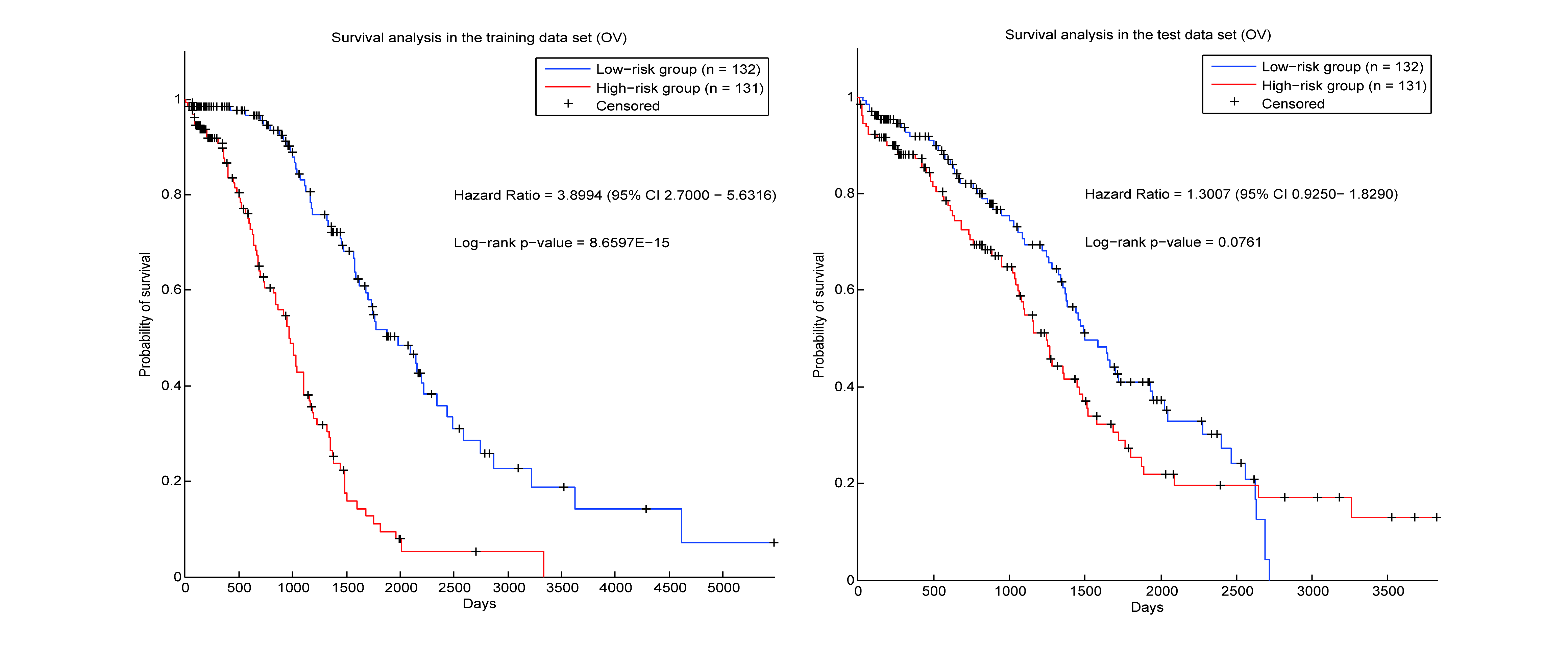


Figure S5. **Survival analysis of ovarian cancer patients divided by the control signature.** **a** The training data set. **b** The test data set.

**
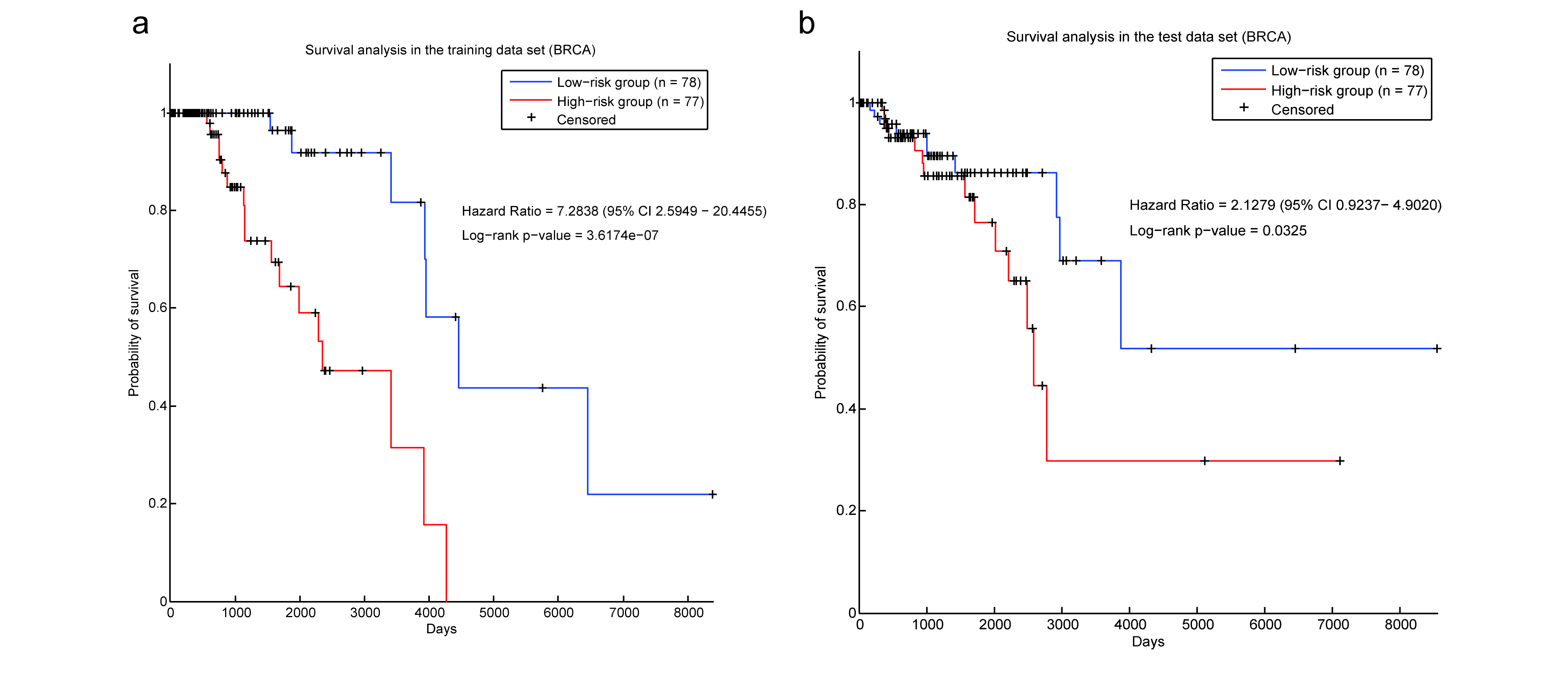
**

Figure S6. **Survival analysis of breast cancer patients divided by the control signature.** **a** The training data set. **b** The test data set.


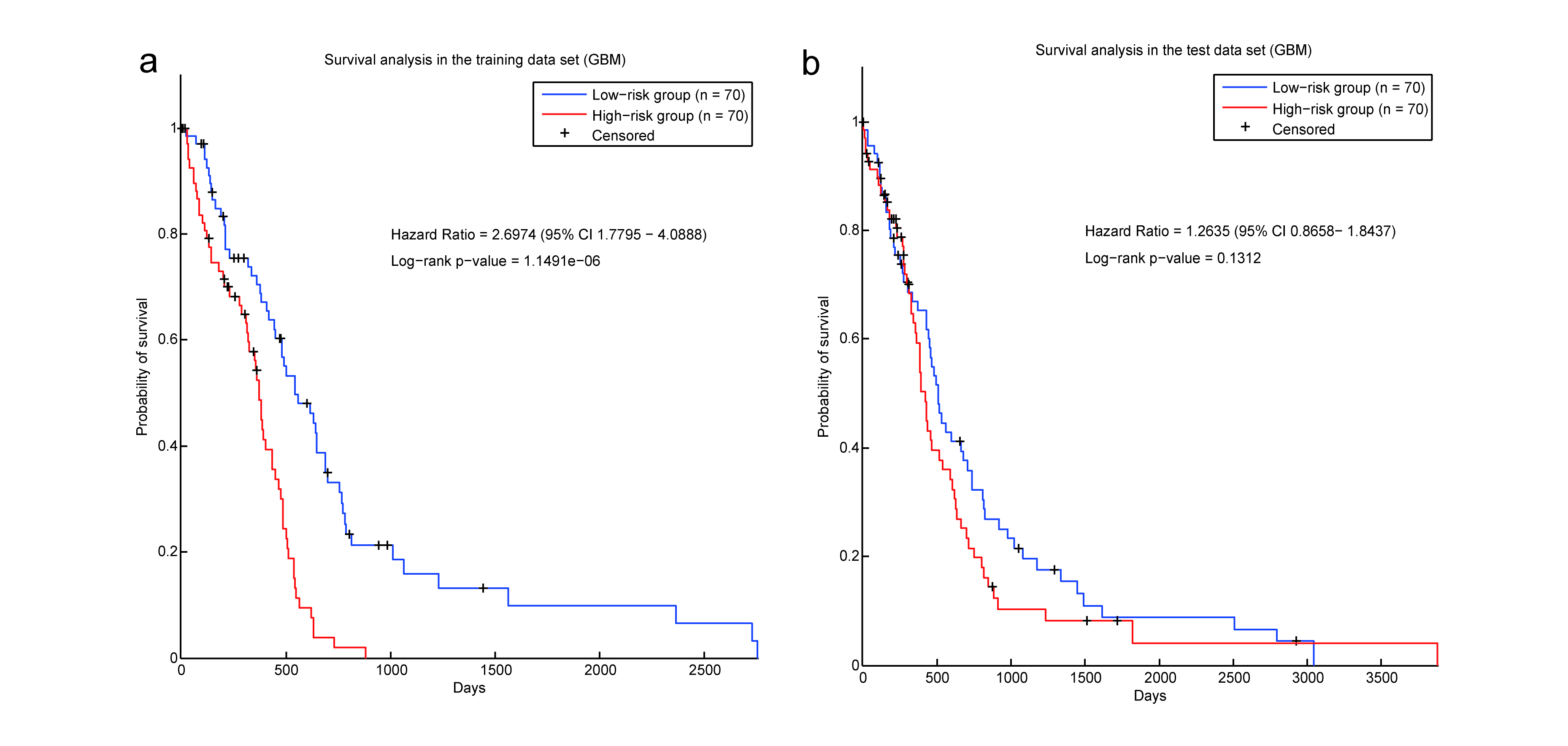


Figure S7. **Survival analysis of cancer patients (glioblastoma multiforme) divided by the control signature.** **a** The training data set. **b** The test data set.

%%%%%%%%%%%%%%%%%%%%%%%%%%%%%%%%%%

function Cnet(ma,DNAm_pro)

%% Construct the DNA methylation interaction network using the rank-based method

%%ma is a m*N matrix, which contain the DNA methylation levels of the samples. m is the number of

%%the DNA methylation sites and N is the number of samples.

%%DNAm_pro is m*1 vector, which contrains the names of the m DNA methylation sites.

ma_r=ma';

N=length(ma_r(1,:));

num=0;

for i=1:N

if sum(ma_r(:,i))~=0

num=num+1;

end;

end;

loc=(1:N)';

res=zeros(num*10,4);

cou=0;

for i=1:N

if sum(ma_r(:,i)~=0) %filter the DNA methylation sites which is zeros across all the samples

cou=cou+1;

[co,p]=corr(ma_r,ma_r(:,i),'type','Spearman'); %%Spearman rank correlation

da=[loc co p];

da=da(~isnan(da(:,2)),:);

da=sortrows(da,-2);

res(((cou-1)*10+1):cou*10,1)=i;

res(((cou-1)*10+1):cou*10,2:4)=da(2:11,1:3); %top 10 nrighbors

end;

end;

cou=1;

cou_max=length(res(:,1));

while cou<=cou_max

result{cou,1}=DNAm_pro{res(cou,1),1};

result{cou,2}=DNAm_pro{res(cou,2),1};

result{cou,3}=res(cou,3);

result{cou,4}=res(cou,4);

cou=cou+1;

end

save('TCGA_DNAm_net.mat','result'); %%Save the network

end
